# Global transcriptome responses including small RNAs during mixed‐species interactions with methicillin‐resistant *Staphylococcus aureus* and *Pseudomonas aeruginosa*

**DOI:** 10.1002/mbo3.427

**Published:** 2016-11-21

**Authors:** Christine L. Miller, Tricia A. Van Laar, Tsute Chen, S. L. Rajasekhar Karna, Ping Chen, Tao You, Kai P. Leung

**Affiliations:** ^1^ Microbiology BranchDental and Craniofacial Trauma Research and Tissue Regeneration DirectorateInstitute of Surgical ResearchJBSA Fort Sam HoustonTXUSA; ^2^ The Forsyth InstituteCambridgeMAUSA

**Keywords:** *Pseudomonas aeruginosa*, sRNA, *Staphylococcus aureus*, transcriptome

## Abstract

*Pseudomonas aeruginosa* and *Staphylococcus aureus* mixed‐species biofilm infections are more resilient to biocide attacks compared to their single‐species counterparts. Therefore, this study used an in vitro model recapitulating bacterial burdens seen in in vivo infections to investigate the interactions of *P. aeruginosa* and *S. aureus* in biofilms. RNA sequencing (RNA‐seq) was utilized to identify the entire genomic response, both open reading frames (ORFs) and small RNAs (sRNAs), of each species. Using competitive indexes, transposon mutants validated uncharacterized PA1595 of *P. aeruginosa* and Panton–Valentine leukocidin ORFs of *S. aureus* are required for competitive success. Assessing spent media on biofilm development determined that the effects of these ORFs are not solely mediated by mechanisms of secretion. Unlike PA1595, leukocidin (*lukS‐PV*) mutants of *S. aureus* lack a competitive advantage through contact‐mediated mechanisms demonstrated by cross‐hatch assays. RNA‐seq results suggested that during planktonic mixed‐species growth there is a robust genomic response or active combat from both pathogens until a state of equilibrium is reached during the maturation of a biofilm. In mixed‐species biofilms, *P. aeruginosa* differentially expressed only 0.3% of its genome, with most ORFs necessary for growth and biofilm development, whereas *S. aureus* modulated approximately 5% of its genome, with ORFs suggestive of a phenotype of increased virulence and metabolic quiescence. Specific expression of characterized sRNAs aligned with the genomic response to presumably coordinate the adaptive changes necessary for this homeostatic mixed‐species biofilm and sRNAs may provide viable foci for the design of future therapeutics.

## 1 INTRODUCTION

The presence of biofilm infections accounts for the healing impairments of 65% of chronic wounds and results in billions of dollars in direct medical costs in the United States (Coenye & Nelis, 2010; Pastar et al., 2013; Rezaei, Safari, Naderinasab, & Aliakbarian, 2011; Seth, Geringer, Galiano, et al., 2012; Seth, Geringer, Hong, et al., 2012; Thomson, 2011). Biofilms are populations of microorganisms that live in a self‐produced matrix of extracellular polymeric substances (EPS) consisting of polysaccharides, proteins, lipids, and extracellular DNA (eDNA) and can be found on biotic and abiotic surfaces (Costerton, Stewart, & Greenberg, 1999; Flemming & Wingender, 2010; Tsuneda, Aikawa, Hayashi, Yuasa, & Hirata, 2003). Biofilms typically comprise diverse microbial species, coordinating together to promote survival in disparate environments. Polymicrobial biofilms lead to impaired wound healing and are more resistant to antibiotic treatment when compared to their monospecies counterparts (Dalton et al., 2011; Madsen, Westh, Danielsen, & Rosdahl, 1996; Pastar et al., 2013; Seth, Geringer, Galiano, et al., 2012; Seth, Geringer, Hong, et al., 2012; Zhao et al., 2010, 2012). Therefore, identifying the cooperative interspecies interactions promoting success and persistence of each species in a biofilm will foster the identification of novel targets for future therapeutics.

The two species found to commonly interact in chronic wounds and other types of infection are *Staphylococcus aureus* and *Pseudomonas aeruginosa* (Baldan et al., 2014; Dalton et al., 2011; Dowd et al., 2008; Joo & Otto, 2012; Kirketerp‐Moller et al., 2008; Michelsen et al., 2014; Pastar et al., 2013; Rezaei et al., 2011; Seth, Geringer, Galiano, et al., 2012; Seth, Geringer, Hong, et al., 2012;Seth et al., 2014). Methicillin‐resistant *S. aureus* (MRSA), a Gram‐positive coccoid bacterium, is responsible for numerous hospital‐ and community‐acquired infections worldwide (Klevens et al., 2007). *Staphylococcus auerus* virulence can be attributed to its ability to evade the host immune system and production of adhesins, toxins, coagulase, and antimicrobial resistance factors (Gordon & Lowy, 2008). *Pseudomonas aeruginosa*, a Gram‐negative bacterium and opportunistic human pathogen, secretes numerous toxic compounds and degradative enzymes (e.g., elastase, LasA protease, phospholipase C, exotoxin A, exoenzyme S, rhamnolipid, hydrogen cyanide [HCN], and pyocyanin) that contribute to pathogenesis (Coggan & Wolfgang, 2012; Van Delden & Iglewski, 1998). To coordinate these virulence mechanisms, both pathogens utilize regulatory networks that comprise noncoding small RNAs (sRNAs) (Gripenland et al., 2010; Sonnleitner, Romeo, & Blasi, 2012; Tomasini et al., 2014). The advent of RNA sequencing has revealed the striking significance of sRNA‐mediated regulation in modulating bacterial lifestyles. However, the role of sRNAs in mixed‐species biofilm interactions between *P. aeruginosa* and *S. aureus* is unknown.

Even though in vivo, *S. aureus* and *P. aeruginosa* are found co‐colonizing, numerous studies have shown that *P. aeruginosa* frequently outcompetes or kills *S. aureus* in vitro (Baldan et al., 2014; Biswas, Biswas, Schlag, Bertram, & Gotz, 2009; Pastar et al., 2013; Seth, Geringer, Galiano, et al., 2012; Seth, Geringer, Hong, et al., 2012). *Pseudomonas aeruginosa*'s ability to outcompete *S. aureus* for the niche have been linked to its ability to sense *S. aureus* through the shedding of peptidoglycan (Korgaonkar, Trivedi, Rumbaugh, & Whiteley, 2013) and secrete numerous products including phenazines (Cardozo et al., 2013), 4‐hydroxy‐2‐heptylquinoline‐*N*‐oxide (Machan, Taylor, Pitt, Cole, & Wilson, 1992), extracellular matrix (Qin, Yang, Qu, Molin, & Tolker‐Nielsen, 2009), and proteases (e.g., LasA and LasB) (Park, Lee, Cho, Herzberg, & Lee, 2012). To survive with *P. aeruginosa* in vivo, *S. aureus* forms small colony variants (Biswas et al., 2009) and induces expression of many virulence factors and regulatory genes (Park et al., 2012). In wounds, it is known that *S. aureus* tends to be found near the surface of the wound, while *P. aeruginosa* is generally found deep in the wound bed (Kirketerp‐Moller et al., 2008), though some studies have shown wounds where morphologically distinct bacteria have segregated into microcolonies with *P. aeruginosa* along the wound margin and other species following behind (Dalton et al., 2011). However, the details of the specific interspecies interactions where they interface are unclear.

Previous studies have addressed gene expression in *P. aeruginosa* (Folsom et al., 2010; Waite et al., 2006) and *S. aureus* (Beenken et al., 2004; Resch et al., 2006) biofilms and have selectively assessed factors thought to contribute to the dynamic state of a mixed‐species biofilm (Park et al., 2012; Pastar et al., 2013). In this study, we have used an in vitro model to investigate the interactions of *P. aeruginosa* and *S. aureus* in biofilm and planktonic cultures and utilized a custom RNA‐sequencing method to unbiasedly capture the global transcriptome response including small RNAs of each species. Since noncoding sRNAs can regulate numerous targets and are capable of controlling a wide range of adaptive processes (Gottesman et al., 2006; Storz, Vogel, & Wassarman, 2011; Tomasini et al., 2014), we hypothesized that regulatory sRNAs play a key role in modulating interspecies interactions in the biofilm. Surprisingly, in our model, *P. aeruginosa* plays a more passive role than previously suspected in the presence of *S. aureus* during biofilm growth by upregulating only a few genes of which most are important for growth and biofilm development. In contrast, *S. aureus* adapts robustly by differentially expressing many genes, including those contributing to the production of capsule and leukocidin, which promotes its success in the dual‐species biofilms and helps *S. aureus* coexist in close proximity with *P. aeruginosa*. Finally, these pathogens express sRNAs that appear to parallel the gene transcription to presumably coordinate the adaptations required for the success of each biofilm species.

## 2 METHODS

### 2.1 Bacterial strains and growth conditions

Strains used are listed in Table 1. Cultures were grown in either 20% or 100% Brain Heart Infusion medium (Thermo Fisher Scientific Remel Products, Lenexa, KS) supplemented with 2% NaCl and 1% glucose (BHI++). For general cell enumeration experiments, cells were plated on trypticase soy agar plates supplemented with 5% sheep's blood (TSA+). For isolation of either *P. aeruginosa* or *S. aureus*, cells were plated on *Pseudomonas* isolation agar (PIA) (Thermo Fisher) or lipovitellin salt mannitol agar (LSMA) (HiMedia Laboratories, Mumbai, India), respectively. Cells were enumerated using the ProtoCOL automated colony counter (Microbiology International, Frederick, MD). A competitive index (CI) was generated by growing a mixture of *P. aeruginosa* and *S. aureus*, seeded together at a 1:1 ratio, and enumerating colony forming units for each species either after reaching mid‐logarithmic growth phase or after 24 hr under biofilm growth conditions. The competitive index was calculated as the *S. aureus*/*P. aeruginosa* output ratio divided by the input inoculum ratio.

**Table 1:** Summary of sequencing reads

Condition	Sequence reads (million)		% Mapping to genome	
	Replicate 1	Replicate 2	*Pseudomonas aeruginosa*	*Staphylococcus aureus*
*P. aeruginosa* biofilm	16.0	16.1	93.9	–
*P. aeruginosa* planktonic	16.2	16.0	95.6	–
*S. aureus* biofilm	18.6	18.1	–	81.2
*S. aureus* planktonic	18.0	17.9	–	84.3
Mixed biofilm	14.5	15.0	48.6	27.8
Mixed planktonic	14.4	13.9	66.1	14.6

### 2.2 Biofilm formation

Biofilms were grown on glass disks in a static system or on glass slides/disks in a drip‐flow biofilm reactor. To grow biofilms on static glass disks, an overnight culture of *P. aeruginosa* or *S. aureus* was diluted 1:20 into fresh 20% BHI++ and grown to an OD_600_ of 0.5. For static biofilms, the culture was then diluted to an OD_600_ of 0.05 into phosphate‐buffered saline (PBS) and incubated with borosilicate glass disks (Ace Glass Inc, Vineland, NJ) in a 24‐well plate with one disk per well for 2 hr at room temperature. For mixed‐species biofilms, a 1:1 mixture of *P. aeruginosa* and *S. aureus* was incubated with the glass disks. After attachment, the disks were rinsed and 1 ml fresh 20% or 100% BHI++ was added to each well. After incubation for 24 hr at 37°C with shaking the disks were rinsed and placed in 1 ml of PBS. Biofilms were detached by sonication and the cells were plated on TSA+, PIA, or LSMA for enumeration after overnight incubation at 37°C. For drip‐flow biofilms, the cultures were prepared as previously mentioned. Following dilution into PBS, 5 ml of cells were injected into each chamber of a drip‐flow reactor (Biosurface Technologies Corporation, Bozeman, MT). Each chamber contained one glass microscope slide and three glass disks. Cells were incubated in the reactor for 2 hr at room temperature at which point the nonadherent cells were drained and the drip‐flow reaction was allowed to proceed with fresh 20% or 100% BHI++ at a rate of 1 ml per minute per chamber for 24 hr at 37°C.

### 2.3 Scanning electron microscopy

Biofilms on borosilicate glass disks were fixed by 2.5% phosphate‐buffered glutaraldehyde for 1 hr at 4°C. The fixed biofilms were dehydrated in a graded series of cold ethanol/water mixture (increasing from 10%, 20%, 30%, 50%, 70%, 80%, 90%, 95% to 100% ethanol) for 6 min each. Critical point dryer EMS 850 (Electron Microscopy Science Hatfield, PA) was used to dehydrate the samples, after which the samples were coated with a gold target using a Hummer 6.2 Sputter Coater (Anatech USA, Hayward, CA). Samples were observed with a Sigma VP40 field emission scanning electron microscope (Carl Zeiss, Inc., Germany) in high vacuum mode at 2 kV (Van Laar, Chen, You, & Leung, 2015).

### 2.4 RNA extraction

Following 24 hr of growth in the drip‐flow reactor, biofilms were removed from the glass slides and preserved in RNA protect (Qiagen). For planktonic cultures, cells were grown to mid‐log phase and harvested by centrifugation. The cells were then preserved in RNA protect. *Staphylococcus aureus* was incubated with lysozyme (Sigma‐Aldrich) for complete cell lysis before RNA extraction. RNA was extracted using the mirVana miRNA Isolation Kit (Life Technologies, Grand Island, NY) according to the manufacturer's instructions for isolation of whole and small RNA fractions. The sample was then treated twice with TURBO DNase (Ambion, Grand Island, NY) according to manufacturer's instructions to remove contaminating genomic DNA. RNA was quantified using a NanoDrop ND‐1000 spectrophotometer (Thermo Scientific, Wilmington, DE) and analyzed for quality using an Agilent 2100 Bioanalyzer (Agilent Technologies, Santa Clara, CA).

### 2.5 RNA sequencing

RNA sequencing was performed by SeqWright (Houston, TX) and the custom strand‐specific sequencing libraries, specifically enriched for small RNAs (<200 bp), were generated as described previously (Gomez‐Lozano, Marvig, Molin, & Long, 2012). Briefly, 2–5 μg of total RNA was used for preparation of both (whole and small RNA) strand‐specific RNA transcriptome sequencing (RNA‐seq) libraries. For whole and small RNA libraries, rRNA, including 5S rRNA, was depleted from total RNA using the Ribo‐Zero Magnetic kit (Epicentre, Madison, WI). The directional RNA‐seq libraries for whole RNA were developed using the NEXTflex directional RNA‐seq (dUTP‐based) kit (Bioo Scientific, Austin, TX). For small RNA libraries specifically, ethanol precipitation was used for clean‐up steps to promote small RNA retention. Depleted RNA from small RNA samples were treated with Tobacco Acid Pyrophosphatase (Epicentre, Madison, WI) at 37°C for 60 min to promote correct adapter ligation followed by organic extraction cleanup (with 25:24:1 phenol:chloroform:isoamyl alcohol) and ethanol precipitation of RNA. Small RNA libraries were prepared using the TruSeq Small RNA sample preparation kit for Adapter ligation (Illumina, San Diego, CA) and sequenced using a paired‐end protocol and read lengths of 100 nucleotides. Both small and whole RNA‐seq libraries were subjected to the quantification process and pooled for cBot amplification and a subsequent sequencing run with a HiSeq 2000 platform (Illumina, San Diego, CA).

After the sequencing run, demultiplexing with CASAVA was employed to generate a FASTQ file for each sample. Single‐end nucleotide reads were mapped to the annotated draft genomic sequence of *P. aeruginosa* PAO1‐UW (GenBank accession no. NC_002516.2) or *S. aureus* subspecies USA300_TCH1516 (GenBank accession no. NC_010079.1) using the software Bowtie (Langmead, Trapnell, Pop, & Salzberg, 2009). The mapped reads were separated into the forward and reverse complement directions using the software tool SAMtools (Li et al., 2009). The mapped reads on each strand were visualized in the JBrowse genome viewer (Skinner, Uzilov, Stein, Mungall, & Holmes, 2009) for sequencing quality.

### 2.6 Differential expression analyses

For differential expression analysis of genes (Table S2 and S3) and small RNAs (Tables S4–S7) raw read counts for the *P. aeruginosa* and *S. aureus* genes were determined with a Perl script based on the mapped read profiles determined above. The read counts were subjected to the Bioconductor software package “DESeq” (Anders & Huber, 2010) to evaluate the differential expression for the RNA between experiments. Two sequencing runs derived from two independently conducted experiments were used in the DESeq analysis. Genes with significantly up‐ or downregulated expression levels were subject to the functional and pathway analysis using CLoVR (Angiuoli et al., 2011) and DAVID software (Huang da et al. 2009, Huang da et al., 2009).

### 2.7 Small RNA detection and categorization

The following steps were carried out to detect and quantify RPKMs (*r*eads *p*er *k*ilobase of transcript per *m*illion mapped reads) for sRNAs (Fig. S2A): (1) combined all four samples (*n* = 2 technical replicates per condition and two conditions compared) read count nucleotide by nucleotide, in both directions separately, and divided by 4; (2) detected seed fragments with an average read count ≥100; (3) extended seed fragments on both sides with read count ≥10; (4) combined extended fragments with gaps ≤5 nt; (5) kept only fragments (sRNAs) that were ≥50nt and ≤500nt in size; (6) calculated RPKM for each sRNA as: ([CountsRNA/SizesRNA] × 1000/CountAll sRNA) × 1,000,000.

Our sRNA classification scheme (Fig. S2B) classified the candidate sRNAs according to functional/structural categories established as described previously (Ferrara et al., 2012; Waters & Storz, 2009). Class I sRNAs located in intergenic regions (>30 nucleotide from flanking ORFs) encompasses *trans*‐encoded sRNAs. Class II5 sRNAs had reads spanning 5′ untranslated regions (5′UTRs) in sense orientation and ≤30 nucleotide overlap would include mRNA riboswitches and sRNAs generated by mRNA transcription attenuation or processing. Class II3 sRNAs had reads spanning 3′ untranslated regions (3′UTRs) in sense orientation and with ≤30 nucleotide overlap. The 3′UTRs are a rich source of sRNAs (Chao, Papenfort, Reinhardt, Sharma, & Vogel, 2012) and would include independently transcribed sRNAs and sRNAs generated by mRNA processing. Class III sRNAs spanning intragenic (<30 nucleotide from flanking ORFs) reads clustering in antisense orientation and *cis*‐encoded antisense sRNAs (asRNAs) would cluster in this class. Class V sRNAs map within ORFs in a sense orientation.

### 2.8 Deletion of *pa1595*

A suicide vector was generated using In‐Fusion HD cloning kit (Clontech Laboratories), as described previously (Miller et al., 2016), which allowed for deletion of the *pa1595* gene through allelic exchange with an antibiotic cassette, pucGM. Briefly, primers were designed according to the manufacturer's suggestion (Table S1) 1 kb upstream and downstream regions of the *pa1595* gene were amplified by PCR from *P. aeruginosa* chromosome. For deletion constructs, a ~1 kb gentamycin antibiotic cassette, pucGM, was PCR amplified from pJQ200. The three fragments for the deletion construct were purified by gel electrophoresis and incubated with the HD‐Infusion enzyme, along with the *Eco*RI linearized pCR2.1 cloning vector and transformed into Stellar™ competent cells. A plasmid with an appropriate insert was identified following restriction enzyme digestion. The deletion vectors were finally linearized using *Eco*RI and electroporated into *P. aeruginosa* to achieve allelic replacement as described previously (Chen & Leung, 2016; Choi, Kumar, & Schweizer, 2006) Two colonies were picked and deletion of the *pa1595* was confirmed by PCR.

### 2.9 Quantitative real‐time PCR analysis

RNA extracted from biofilms and planktonic cultures was converted to cDNA using the iScript Select cDNA Synthesis Kit (Bio‐Rad, Hercules, CA). cDNA was subjected to quantitative real‐time PCR using SYBR Green PCR master mix (Bio‐Rad) with a final concentration of 0.3 μmol/L of oligonucleotides (Table S1) using the ABI Prism 7300 system (Applied Biosystems) (Van Laar et al., 2015). Gene expression levels were compared for selected hits generated from RNA sequencing results. Data were normalized to expression levels of *gmk* for *S. aureus* RNA and *fabD* for *P. aeruginosa* RNA.

### 2.10 Spent media preparation and testing

Planktonic cultures of *P. aeruginosa* or *S. aureus* were grown overnight in 20% BHI++ at 37°C with shaking. The cultures were centrifuged for 30 min at 4,000*g* (Childers, Van Laar, You, Clegg, & Leung, 2013), and supernatants recovered were filtered sterilized using a 0.22‐μm syringe filter and used immediately. Heat‐treated supernatants were prepared by boiling filtered supernatants for 15 min. Spent media supernatants were added to 100% BHI++ to make a final concentration of 20% BHI++ for growth. To measure the effects of spent media on biofilm formation, biofilms were prepared as above in each well of a 96‐well plate. Biofilm viability was determined using the PrestoBlue Cell Viability Reagent (Life Technologies) according to the manufacturer's instructions. Briefly, biofilms were rinsed three times with PBS and incubated with a 1:10 dilution of PrestoBlue in 20% BHI++ for 30 min at 37°C. Excitation and emission of PrestoBlue were read at 535 nm and 590 nm, respectively.

### 2.11 Swarming assays

Swarming motility assays were performed as described previously (Heurlier et al., 2004). Bacteria were grown in 20% BHI++ medium overnight for 16 hr and 3 μl of culture was plated in four independent replicates. Swarming was evaluated on plates containing 0.5% w/v Bacto agar (Difco), 8 g of nutrient broth (Oxoid) per liter, and 0.5% w/v d‐glucose. Swarming was observed after 24 hr of incubation at 37°C and was repeated on three separate occasions.

### 2.12 Pyocyanin (PYO) assay

PYO quantitation was performed as described previously (Essar, Eberly, Hadero, & Crawford, 1990). Specifically, a 5‐ml sample of culture grown overnight in 20% BHI++ was centrifuged at 12,000*g* for 20 min and the supernatant was extracted with 3 ml of chloroform and then re‐extracted into 1 ml of 0.2 N HCl to give a pink to deep red solution. The absorbance of this solution was measured at 520 nm.

### 2.13 Cross‐streak assay

A cross‐streak assay was performed as described previously (Michelsen et al., 2014). Briefly, one loopful of an overnight culture of *P. aeruginosa* was streaked horizontally across TSA+ plates and allowed to dry. One loopful of an overnight culture of *S. aureus* was then streaked vertically on the same plate. Once the *S. aureus* streak dried, the plates were incubated overnight at 37°C. The plates were then photographed to allow qualitative interpretation of the *P. aeruginosa*–*S. aureus* interaction.

### 2.14 Statistical analysis

All statistical data were calculated using GraphPad Prism version 5.0 for Macintosh (GraphPad Prism Software, San Diego, CA). Statistical significance was accepted when *P* values were equal to or less than 0.05.

### 2.15 RNA‐seq data accession number

The RNA‐seq reads and the DESeq results have been deposited in NCBI's Gene Expression Omnibus (Edgar, Domrachev, & Lash, 2002) and are accessible through Bioproject accession no PRJNA341717.

## 3 RESULTS

### 3.1 In vitro growth conditions mimic *P. aeruginosa* and *S. aureus* burdens seen in dual‐species biofilms of the wound environment

To determine which culture conditions would best mimic the wound environment and bacterial burden ratios seen in wound infections, we grew our cultures in two different concentrations of BHI++ (20% and 100%), a chopped meat‐based media.

**Figure 1:**
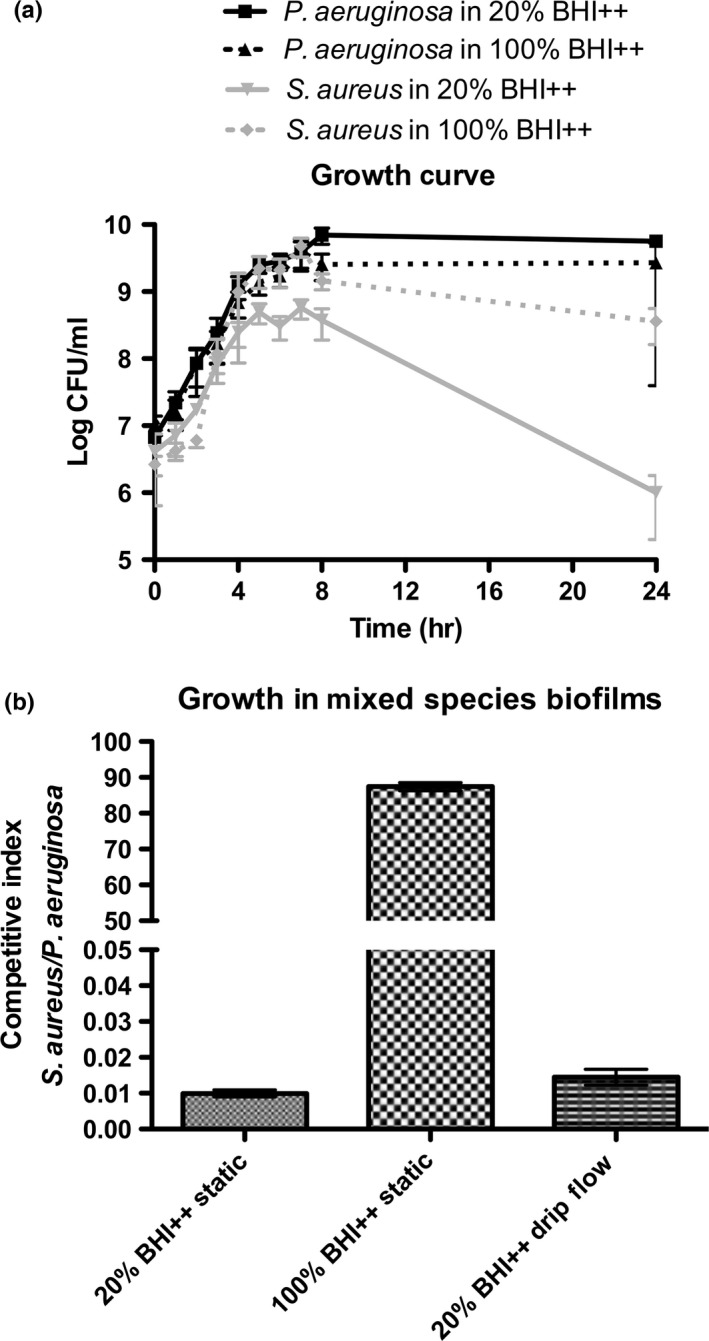
In vitro growth conditions to mimic bacterial burden ratios seen in in vivo mixed‐species infections. *Staphylococcus aureus* grown in 100% BHI++ with *Pseudomonas aeruginosa* competes better and grows to higher numbers compared to coculture planktonic growth in 20%BHI++, where after 24 hr *P. aeruginosa* outcompetes *S. aureus*. Cell enumeration using colony‐forming units depicts *P. aeruginosa* and *S. aureus* growth dynamics when seeded together at a 1:1 ratio and grown under planktonic growth conditions in either 20% or 100% BHI++ media (a). *Pseudomonas aeruginosa* and *S. aureus* subcultures were seeded together at a 1:1 ratio and grown under static or drip‐flow biofilm conditions in either 20% or 100% BHI++ media. Unlike 100% BHI++, growth as either a static or drip‐flow mixed‐species biofilm in 20% BHI++ media yields bacterial ratios previously seen in in vivo infections; specifically, a *P. aeruginosa* to *S. aureus* ratio of approximately 100:1. A competitive index (CI) was calculated as the *S. aureus* CFU/*P. aeruginosa* CFU output ratio divided by the input inoculum ratio (b). Data represent the mean ± standard deviation from at least four independent experiments

Using 100% BHI++ for the growth of mixed planktonic cultures, we found that when starting with a bacterial ratio of 1:1, there was little difference during log phase, but after 24 hr, *P. aeruginosa* outcompeted *S. aureus* by about one log (Figure 1a). Mixed planktonic cultures grown in 20% BHI++ had similar bacterial ratios as cultures grown in 100% BHI++ during early growth phases, but after 24 hr, *S. aureus* numbers dropped significantly (Figure 1a). When growing mixed biofilms, we used direct cell numbers to calculate a competitive index (CI). The CI was calculated as the *S. aureus*/*P. aeruginosa* output ratio divided by the input inoculum ratio; therefore, a CI greater than 1 indicates that *S. aureus* is in greater numbers in the dual‐species biofilm. Biofilms grown under static conditions in 100% BHI++ yielded a CI of 87.4 with *S. aureus* strongly outcompeting *P. aeruginosa*; whereas growth in 20% BHI++ yielded a CI of 9.9 × 10^−3^ (Figure 1b), representing approximately 100 *P. aeruginosa* for every 1 *S. aureus*, a similar ratio previously reported in a porcine wound model (Pastar et al., 2013). Therefore, we chose to use 20% BHI++ for further experiments. To obtain sufficient quantities of RNA for sequencing, biofilms were grown under drip‐flow conditions because the CI of biofilms grown in the drip‐flow was not significantly different from the CI of static biofilms grown in 20% BHI++ (CI 1.4 × 10^−2^, *p *=* *.14) (Figure 1b).

### 3.2 Scanning electron microscope demonstrates *P. aeruginosa* and *S. aureus* growth dynamics and burden in in vitro dual‐species biofilms

**Figure 2:**
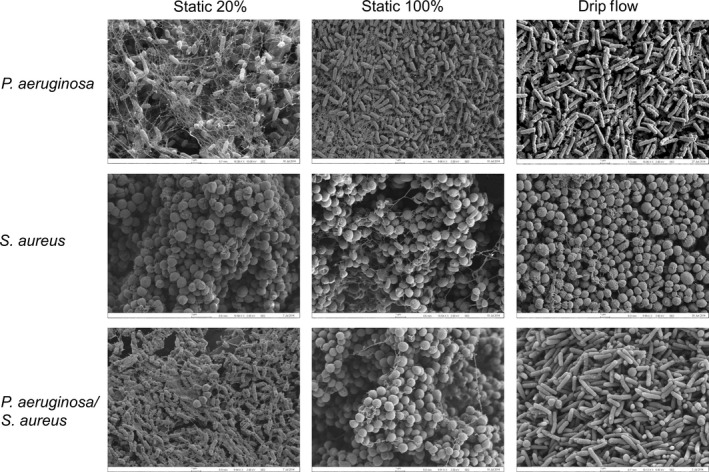
SEM micrograph depicts similar trends in the ratios of *Pseudomonas aeruginosa* and *Staphylococcus aureus* in mixed‐species cocultures as previously observed using direct cell counting in Figure 1. Single‐ and mixed‐species biofilms were grown in 20% BHI++ under static and drip‐flow conditions and 100% BHI++ under static conditions. In mixed cultures grown with 20% BHI++ (static or drip‐flow), *P. aeruginosa* was the dominant species. In static biofilms grown in 100% BHI++, *S. aureus* significantly outcompetes *P. aeruginosa*. Images show 10,000× magnification and are representative of three independent experiments

Single‐ and mixed‐species biofilms were grown in 20% BHI++ under static and drip‐flow conditions and 100% BHI++ under static conditions. The mixed‐species biofilm micrographs depicted trends corresponding to the ratios observed using direct cell counting (Figure 2). In both static and drip‐flow biofilms grown in 20% BHI++, *P. aeruginosa* significantly outcompeted *S. aureus*, while the opposite trend was seen with biofilms grown in 100% BHI++ under static conditions.

### 3.3 Differential gene expression of *P. aeruginosa* and *S. aureus* in mixed biofilm or planktonic cultures when compared to single‐species conditions

RNA sequencing was performed on RNA isolated from mixed‐ and single‐species biofilm and planktonic cultures (collected at an OD_600_ of 0.5) of *P. aeruginosa* and *S. aureus*. Two replicates of each experimental conditional were assessed and gene expression trends were confirmed using quantitative real‐time PCR (qRT‐PCR) for randomly chosen genes with high levels of differential expression with RNA‐seq (Fig. S1). The read numbers generated from each of the libraries of repeats are as follows: *P. aeruginosa* single‐species biofilm, 16.0 and 16.1 million sequence reads; *P. aeruginosa* single‐species planktonic, 16.2 and 16.0 million sequence reads; *S. aureus* single‐species biofilm, 18.6 and 18.1 million sequence reads; *S. aureus* single‐species planktonic, 18.0 and 17.9 million sequence reads; mixed‐species biofilms, 14.5 and 15.0 million sequence reads; and mixed‐species planktonic cultures, 14.4 and 13.9 million sequence reads. For single‐species *P. aeruginosa* cultures, an average of 95% of reads was mapped to the MPAO1 genome. For single‐species *S. aureus* cultures, an average of 83% of reads was mapped to the USA300HOU genome. When considering mixed‐species planktonic cultures, an average of 66% of reads was mapped to the MPAO1 genome, while an average of 15% of reads was mapped to the USA300HOU genome. In mixed‐species biofilms, an average of 48% of reads was mapped to the MPAO1, while an average of 28% of reads was mapped to the USA300HOU genome. The RNA sequence reads and mapping are summarized in Table 1.

Table 2 contains a list of the top 10 upregulated and downregulated ORFs in *P. aeruginosa* and *S. aureus* when grown in a mixed‐ versus single‐species biofilm. Using a ≥2 fold change and *p* value ≤.05 cutoff, *P. aeruginosa* grown in mixed‐species biofilms demonstrated nine upregulated and seven downregulated ORFs compared to those grown in single‐species biofilms (Table 2 and S2). When comparing *P. aeruginosa* grown in mixed‐ versus single‐species planktonic cultures, *P. aeruginosa* up‐ and downregulated approximately 1,000 and 1,300 ORFs, respectively. Since there were over 2,000 *P. aeruginosa* ORFs differently expressed in mixed‐ versus single‐species planktonic cultures, for further data analysis of this comparison only, we analyzed only those ORFs with a fold change ≥5, resulting in 103 up‐ and 330 downregulated ORFs (Table S2).

**Table 2:** Top open reading frames differentially expressed under mixed biofilm conditions

Organism	Gene ID	Annotation	Log2 expression change
*Pseudomonas aeruginosa*	PA1632	Potassium‐transporting ATPase F	4.51
	PA3432	Hypothetical protein	3.71
	PA3431	Hypothetical protein	3.41
	PA1854	Hypothetical protein	3.01
	PA0529	Hypothetical protein	2.89
	PA4220	Hypothetical protein	2.76
	PA0530	Class III pyridoxal phosphate‐dependent aminotransferase	2.76
	PA3914	Molybdenum cofactor biosynthetic protein A1	2.75
	PA3405	Metalloprotease secretion protein	2.75
	PA0531	Glutamine amidotransferase	2.56
	PA1092	Flagellin type B	−1.96
	PA2015	Isovaleryl‐CoA dehydrogenase	−2.68
	PA3841	Exoenzyme S	−2.72
	PA1978	Response regulator ErbR	−2.78
	PA4211	Phenazine biosynthesis protein	−2.79
	PA0865	4‐hydroxyphenylpyruvate dioxygenase	−3.02
	PA2000	Dehydrocarnitine CoA transferase subunit B	−3.08
*Staphylococcus aureus*	USA300HOU_2200	Hypothetical protein	3.01
	USA300HOU_2551	Hypothetical protein	2.67
	USA300HOU_0130	Major facilitator transporter	2.55
	USA300HOU_0128	Ornithine cyclodeminase	2.52
	USA300HOU_1382	Hypothetical protein	2.45
	USA300HOU_0129	IucA/IucC family siderophore biosynthesis protein	2.41
	USA300HOU_0127	Pyridoxal phosphate‐dependent enzyme	2.39
	USA300HOU_0572	Hypothetical protein	2.32
	USA300HOU_1065	Iron (Fe2+)‐regulated surface determinant protein IsdC	2.20
	USA300HOU_0955	Transcriptional regulator Spx	2.13
	USA300HOU_1138	Aspartate carbamoyltransferase catalytic subunit	−2.55
	USA300HOU_1137	Uracil permease	−2.65
	USA300HOU_1012	Phosphoribosylformylglycinamidine synthase	−2.66
	USA300HOU_0841	Hypothetical protein	−2.70
	USA300HOU_1016	Phosphoribosylaminoimidazole synthetase	−2.73
	USA300HOU_1014	Phosphoribosylformylglycinamidine synthase II	−2.78
	USA300HOU_1015	Amidophosphoribosyltransferase	−2.90
	USA300HOU_1013	Phosphoribosylformylglycinamidine synthase I	−3.08
	USA300HOU_0190	Formate dehydrogenase	−3.35
	USA300HOU_0309	Hypothetical protein	−3.53

*Staphylococcus aureus* had 79 up‐ and 63 downregulated ORFs when comparing mixed‐ versus single‐species biofilms (Table S3). In a mixed planktonic culture, there were 150 *S. aureus* ORFs upregulated and 115 *S. aureus* ORFs downregulated when compared to a *S. aureus* single‐species planktonic culture (Table S3). Table 3 contains a summary of these results, in which each ORF was assigned to an orthologous group (COG) based on predicted or known function (Angiuoli et al., 2011; Winsor et al., 2011).

**Table 3:** Differentially expressed open reading frames (ORFs) organized by cluster of orthologous groups of proteins (COGs) category

ORF category by COG function (s)	No. (%) ORFs with change in expression for^a^							
	Mixed versus *Pseudomonas aeruginosa* planktonic		Mixed versus *P. aeruginosa* biofilm		Mixed versus *Staphylococcus aureus* planktonic		Mixed versus *S. aureus* biofilm	
	Upregulated	Downregulated	Upregulated	Downregulated	Upregulated	Downregulated	Upregulated	Downregulated
Cellular processes and signaling								
Cell cycle control, cell division, chromosome partitioning		1 (0.3)			1 (0.7)			
Cell wall, membrane, and envelope biogenesis	5 (4.9)	7 (2.1)	1 (11.1)		1 (0.7)	3 (2.6)	13 (16.5)	1 (1.6)
Cell motility	4 (3.9)	1 (0.3)		1 (14.3)	1 (0.7)			
Post‐translational modification, protein turnover, and chaparones	2 (1.9)	15 (4.5)			2 (1.3)	1 (0.9)	1 (1.3)	
Signal transduction mechanisms	3 (2.9)	6 (1.8)	1 (11.1)	2 (28.6)	3 (2.0)			
Intracellular trafficking, secretion, and vesicular transport	7 (6.8)	15 (4.5)			1 (0.7)			
Extracellular structures					1 (0.7)		11 (13.9)	
Defense mechanisms	2 (1.9)	7 (2.1)	1 (11.1)	1 (14.3)	10 (6.7)	7 (6.1)	6 (8.0)	6 (9.5)
Information storage and processing								
Translation, ribosomal structure and biogenesis	1 (1.0)	39 (11.8)			7 (4.7)	25 (21.7)	1 (1.3)	2 (3.2)
Transcription	4 (3.9)	5 (1.5)		1 (14.3)	11 (7.3)	4 (3.5)	4 (5.1)	
Replication, recombination, and repair	2 (1.9)	2 (0.6)			3 (2.0)	7 (6.1)		
Metabolism								
Energy production and conversion	5 (4.9)	40 (12.1)			12 (8.0)	11 (9.6)	7 (8.9)	7 (11.1)
Amino acid transport and metabolism	10 (9.7)	51 (15.5)	1 (11.1)	1 (14.3)	10 (6.7)	23 (20.0)	11 (13.9)	18 (28.6)
Nucleotide transport and metabolism	5 (4.9)	10 (3.0)	1 (11.1)		4 (2.7)	12 (10.4)	2 (2.5)	22 (34.9)
Carbohydrate transport and metabolism	4 (3.9)	13 (3.9)			7 (4.7)	1 (0.9)	13 (16.5)	
Coenzyme transport and metabolism	3 (2.9)	10 (3.0)	1 (11.1)			1 (0.9)		
Lipid transport and metabolism	4 (3.9)	15 (4.5)		2 (28.6)	1 (0.7)		2 (2.5)	
Inorganic ion transport and metabolism	9 (8.7)	14 (4.2)	1 (11.1)		4 (2.7)	15 (13.0)	9 (11.4)	7 (11.1)
Secondary metabolites biosynthesis, transport, and catabolism	3 (2.9)	4 (1.2)			2 (1.3)	3 (2.6)	5 (6.3)	
Poorly characterized								
General function prediction only	7 (6.8)	21 (6.4)	1 (11.1)	1 (14.3)	12 (8)	9 (7.8)	3 (3.8)	2 (3.2)
Function unknown	35 (34.0)	71 (21.5)	2 (22.2)		66 (44)	10 (8.7)	22 (27.8)	4 (6.3)

a Percentages total more than 100% due to ORFs belonging to multiple functional categories.

### 3.4 *Pseudomonas aeruginosa*

#### 3.4.1 Few genes are differentially expressed in *P. aeruginosa* when grown in a biofilm with *S. aureus*

Using our criteria for determining significant differential gene expression (≥2 fold change, *p *<* *.05), only 16 ORFs (nine up‐ and seven downregulated) experienced expression changes when *P. aeruginosa* was grown in a mixed‐ versus single‐species biofilm (Table 3). Three of the downregulated ORFs (*exoS/PA3841*,* phzB1/PA4211*, and *fliC/PA1092*) are components of important *P. aeruginosa* virulence factors. The protein products of *exoS* and *phzB1* are toxic molecules that allow for invasion and killing of the host (Cezairliyan et al., 2013; Rangel, Diaz, Knoten, Zhang, & Hauser, 2015). Also, both ExoS and FliC are secreted by the type III secretion system and play important roles in stimulating the host inflammatory response (Ince, Sutterwala, & Yahr, 2015). The upregulated ORFs were categorized as being involved in general cell maintenance functions such as ion transport and utilization (e.g., *moeA/PA3914*,* hasE/*,* kdpE/PA1632*,* PA4220*, and *PA1854*) and amino acid metabolism (Table 2). One study demonstrated that amino acid metabolism along with cell motility and stress response is implicated in biofilm development in *Salmonella enterica* serovar Typhimurium (Hamilton et al., 2009).

Of the few ORFs upregulated in *P. aeruginosa* during mixed‐species biofilm growth were *PA3431* (>11‐fold, *p *<* *1.8e−5, Table 2) and *PA3432* (approximately 13‐fold, *p *<* *1.4e−3). Interestingly, PA3431and PA3432 are orthologs to CidB/LrgB and CidA/LrgA, respectively. CidB/LrgB in conjunction with CidA/LrgA are part of the holin/antiholin system that promotes and protects from cell lysis by murein hydrolyases (Groicher, Firek, Fujimoto, & Bayles, 2000). Resulting cell lysis and release of cellular components provides material for the structural components of the biofilm (Ma et al., 2009; Rice et al., 2007).

#### 3.4.2 *Pseudomonas aeruginosa* grown in mixed planktonic cultures downregulates many ORFs involved in metabolism, general cellular processes, and type VI secretion

In contrast to the limited set of ORFs that we found to be differentially expressed when *P. aeruginosa* was grown in a mixed‐species biofilm, hundreds of ORFs were differentially expressed when *P. aeruginosa* was grown in a mixed planktonic culture (Tables 3 and S2). Using our revised criteria of ≥5 fold change, *p *<* *.05, one of the more obvious trends observed was significant downregulation of ORFs involved in amino acid transport and metabolism (51 ORFs; 15.5% of differentially expressed ORFs) as well as energy production (40 ORFs; 12.1% of differentially expressed ORFs). Because amino acid transport and metabolism could be involved in biofilm formation (Hamilton et al., 2009), therefore, downregulation of these ORFs might negatively impact biofilm development. There was also downregulation of numerous ORFs encoding ribosomal proteins, indicating an overall decrease in translation. Approximately 3% of the most prominent downregulated ORFs are involved in the type VI secretion system (T6SS) (*PA0074* to *PA0091*). *Pseudomonas aeruginosa* uses its T6SS to secrete toxins (Tse1–3) into competing bacteria that hydrolyze peptidoglycan (Hood et al., 2010; Russell et al., 2011). To protect itself from these toxins, *P. aeruginosa* synthesizes immunity proteins Tsi1 and Tsi3 that were also downregulated along with the toxins.

Interestingly, when *P. aeruginosa* is grown as a planktonic culture with *S. aureus*, 34% of the most prominent upregulated ORFs have unknown functions which promotes speculation for their role in coordinating events allowing *P. aeruginosa* to dominate during the later stage of planktonic growth.

### 3.5 *Staphylococcus aureus*

#### 3.5.1 *Staphylocccus aureus* downregulates amino acid and nucleotide biosynthesis when grown with *P. aeruginosa* in biofilms

Differential gene expression analysis showed a marked decrease in the expression of amino acid and nucleotide biosynthetic genes in *S. aureus* when comparing mixed‐ and single‐species biofilms (Table 2 and S3). While there were two ORFs involved in lysine and arginine biosynthesis upregulated, the trend clearly favors downregulation of amino acid metabolism. Along those same lines, there were two ribonucleotide reductase proteins (involved in DNA biosynthesis) upregulated. However, nearly 20 ORFs (13.4% of total differentially expressed ORFs) involved in purine and pyrimidine biosynthesis were downregulated when *S. aureus* was grown in a mixed‐species biofilm compared to a single‐species biofilm. Similar to *P*. *aeruginosa*, the downregulation of ORFs that were involved in amino acid metabolism in *S. aureus* could play a role in decreasing biofilm formation of *S. aureus* in the mixed‐species settings (Hamilton et al., 2009).

#### 3.5.2 Various *S. aureus* virulence factors are differentially expressed in mixed biofilms

RNA‐seq analysis showed upregulation of known staphylococcal virulence factors including Panton–Valentine leukocidin (*lukS‐PV* and *lukF‐PV*), protein A (*spA*), a protease (*clp*), and a complement inhibitor. Many of these proteins are secreted factors that facilitate host cell destruction and immune evasion (Lina et al., 1999; Zhang, Jacobsson, Vasi, Lindberg, & Frykberg, 1998). Iron sequestration genes belonging to the Isd family (including *isdA*,* C*,* E*, and *G2*) were upregulated when *S. aureus* was grown in a biofilm with *P. aeruginosa*. This family is involved in acquiring heme for scavenging iron (Tiedemann, Muryoi, Heinrichs, & Stillman, 2008). In addition, three proteins belonging to the IucA/IucC family of siderophore biosynthesis were upregulated. These proteins are important for *S. aureus* to acquire iron (de Lorenzo & Neilands, 1986), an element *P. aeruginosa* is known to acquire from *S. aureus* in coculture (Mashburn, Jett, Akins, & Whiteley, 2005). Capsule biosynthetic genes (*cap5EF*,* I‐P*) were also upregulated under mixed biofilm conditions. Capsule is known to be an important virulence factor that allows the cells to both adhere to host tissues and avoid phagocytosis (Li et al., 2014), though USA300 derivative strains (such as TCH1516 and JE2) have been shown to be lacking in capsule production (Boyle‐Vavra et al., 2015). However, RNA‐seq revealed downregulation of other virulence factors including fibronectin‐binding protein B (*fnbB*), clumping factor B (*clfB*), staphylocoagulase (*coa*), and a superantigen‐like protein (*set*). Both FnbB and ClfB are involved in adhesion and invasion of host connective tissue (McCourt, O'Halloran, McCarthy, O'Gara, & Geoghegan, 2014; O'Brien, Walsh, Massey, Peacock, & Foster, 2002). *Coa* causes blood clotting in infected hosts and is important for biofilm formation (Friedrich et al., 2003; Zapotoczna, McCarthy, Rudkin, O'Gara, & O'Neill, 2015) while *set* (and the other superantigen‐like proteins) bind complement factors and the Fc regions of antibodies (Hermans et al., 2012).

#### 3.5.3 *Staphylocccus aureus* downregulates various metabolic processes when grown in planktonic cultures with *P. aeruginosa*

Similar to the trends observed when *S. aureus* is grown in a biofilm with *P. aeruginosa*, under mixed planktonic conditions, there is an overall decrease in expression of genes whose products are necessary for amino acid transport and metabolism. As with mixed biofilm conditions, the two ribonucleotide reductase genes were upregulated while the majority of the rest of the nucleotide transport and metabolism ORFs were downregulated. In addition, there was a downregulation of a number of ORFs involved in translation, including 13 ribosomal proteins and a handful of tRNA synthesis proteins.

#### 3.5.4 There is differential expression of stress‐response genes and virulence factors when *S. aureus* is grown in mixed planktonic cultures

Consistent with mixed biofilm conditions, there was a downregulation of *fnbB* and *coa* in a mixed planktonic culture. Two ORFs predicted to encode enterotoxins (USA300HOU_0851 and USA300HOU_0852) and staphopain (USA300HOU_1910), a peptidase whose targets include elastin, were also downregulated under these conditions. Contrary to mixed biofilm conditions, RNA‐seq of mixed planktonic cultures revealed an upregulation of two superantigen‐like proteins. The gene encoding staphylokinase (*sak*) was also upregulated. *Sak* is known to have a role in allowing invasiveness in the skin and has recently been shown to inhibit biofilm formation (Kwiecinski et al., 2015), however a regulator promoting biofilm formation (*rot*) (Mootz et al., 2015) was also upregulated.

Two ORFs involved in the oxidative stress response (*ahpC1, ahpF*) (Poole et al., 2000) were downregulated, while genes responsible for nitrate respiration (*narHJKL*) and reduction (*nirBD*) normally activated under anaerobic growth conditions (Fuchs, Pane‐Farre, Kohler, Hecker, & Engelmann, 2007) were upregulated in mixed planktonic cultures. *Staphylococcus aureus* also upregulated *epiA*, a gene responsible for the production of the lantibiotic epidermin, though a putative transporter of a different lantibiotic, salivaricin A, *salX* was downregulated. Although lantibiotics are effective only against other Gram‐positive organisms (Sahl & Bierbaum, 1998), their production indicates a defensive strategy by *S. aureus* when grown in competition. Finally, there was upregulation of two genes implicated in antibiotic resistance, *lrgA* (which is also part of a holin/antiholin system) (Groicher et al., 2000) and a tellurite transporter (Taylor, 1999).

### 3.6 Novel ORFs contribute to competitive success

#### 3.6.1 Specific ORFs upregulated under mixed‐species biofilm conditions contribute to competitive success of either *S. aureus* or *P. aeruginosa*

To determine the contributions of the ORFs upregulated in either *P. aeruginosa* or *S. aureus* when grown under mixed‐species biofilm conditions, transposon mutants deficient of these ORFs were grown in dual‐species biofilms with wild‐type competitors and colony‐forming units of each species after 24 hr growth were assessed to generate a competitive index (CI). Before a CI could be obtained for the mutants, growth curves and biofilm assays were performed for each transposon mutant to ensure that any defect in competition could not be attributed to deficiencies in growth or ability to form biofilms. Any mutant found to be defective in growth or biofilm formation was not considered for future downstream analysis.

**Figure 3:**
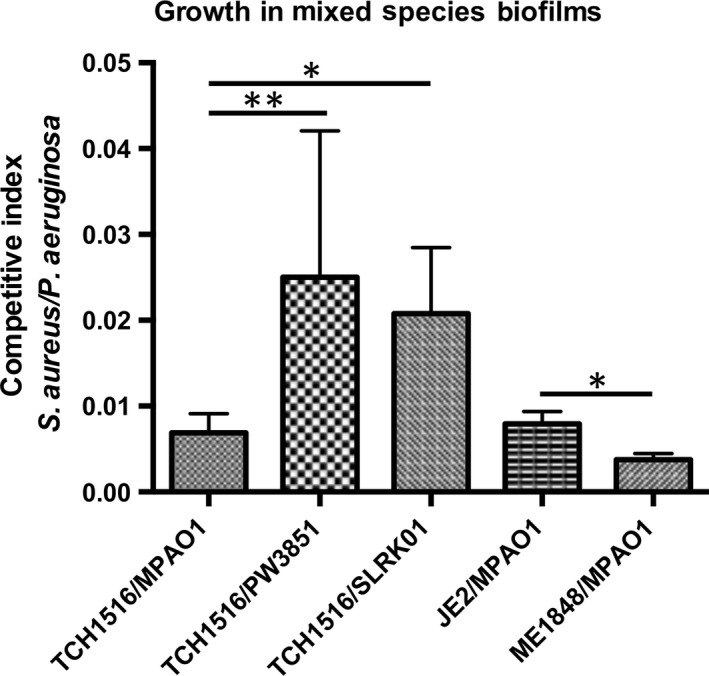
Mutants corresponding to open reading frames upregulated in either *Staphylococcus aureus* or *Pseudomonas aeruginosa* during mixed‐species biofilms were defective for competition in mixed‐species biofilms. An overnight culture of *P. aeruginosa* or *S. aureus* was diluted into fresh 20% BHI++ and allowed to reach an OD_600_ of 0.5, before seeding the bacteria at a 1:1 ratio for attachment and maturation into a 24‐hr static mixed‐species biofilm. Biofilms were detached by sonication and the cells were plated on selective media for enumeration. A competitive index (CI) was calculated as the *S. aureus* CFU/*P. aeruginosa* CFU output ratio divided by the input inoculum ratio. The *PA1595* transposon mutant (strain PW3851) and clean knockout (strain SLRK01) were defective for competition allowing a tip in the CI favoring more *S. aureus* growth compared to the mixed‐species biofilm established with wild‐type strains (TCH1516/MPAO1). The *lukS‐PV* (strain NE1848) transposon mutant derived from parental *S. aureus* strain JE2 was defective for competition with a CI ratio skewed to favor more *P. aeruginosa* growth compared to the mixed‐species biofilm established with wild‐type strains (JE2/MPAO1). Data represent the mean ± standard deviation from at least four independent experiments. Significance was determined by unpaired Student's *t*‐test (one‐tailed) (*p <* *.05; **p <* *.01)

All the ORFs upregulated in *P. aeruginosa* in mixed‐species biofilms listed in Table 2 were also important for either growth or biofilm development, and were thus unsuitable for further analysis through competition assays. We chose an upregulated gene, *PA1595*, which was upregulated fourfold (Table S2) in wild‐type *P. aeruginosa* grown in mixed‐species biofilms to characterize in terms of its contribution to competitive ability. PA1595 mutant displayed no growth defects. PA1595 is a hypothetical protein predicted to localize to the outer membrane, with no identified conserved domains. The CI of a biofilm consisting of the *PA1595* transposon mutant (strain PW3851) and wild‐type *S. aureus* was 2.5 × 10^−2^, 2.5‐fold higher than the CI of a biofilm consisting of parental strains (TCH1516/MPAO1; CI = 9.9 × 10^−3^, *p* *<* .01), while the CI of the clean *PA1595* knockout (strain SLRK01) was 2.1 × 10^−2^ (*p *<* *.05) (Figure 3) suggesting that the lack of PA1595 allows for *S. aureus* to be more abundant in a mixed‐species biofilm and thus important for *P. aeruginosa* to compete with *S. aureus* in the biofilm niche.

The *S. aureus* transposon mutant library was generated in JE2 background (Fey et al., 2013). The CI between JE2 and *P. aeruginosa* was insignificant from the CI of MPAO1 and TCH1516 (CI = 9.26 × 10^−3^, *p = *.3). *Staphylococcus aureus lukS‐PV*, a component of the Panton–Valentine leukocidin, were upregulated 2.3‐fold when *S. aureus* was grown in a mixed‐species biofilm. Compared to the CI of the parental strains, the transposon mutant *lukS‐PV* (strain NE1848) was significantly impaired in their ability to compete with *P. aeruginosa* in a mixed biofilm as evidenced by lower CI value (3.4 × 10^−3^, *p *<* *.05), which is 2.7‐fold lower than the CI of the parental strains (Figure 3). These data suggest that leukocidin plays a role in the ability of *S. aureus* to compete with *P. aeruginosa* in mixed‐species biofilms.

#### 3.6.2 The inability of mutant *S. aureus* and *P. aeruginosa* to compete in a biofilm is not attributed to an inability to combat the competitor's exoproducts

We sought to characterize the mechanisms contributed by these ORFs leading to a decrease in the ability of the aforementioned mutants to compete. Because *P. aeruginosa* and *S. aureus* interact indirectly by exposure to secreted products, we assessed how these ORFs might influence mixed‐species biofilm dynamics by determining the contribution of spent media on select mutants.

**Figure 4:**
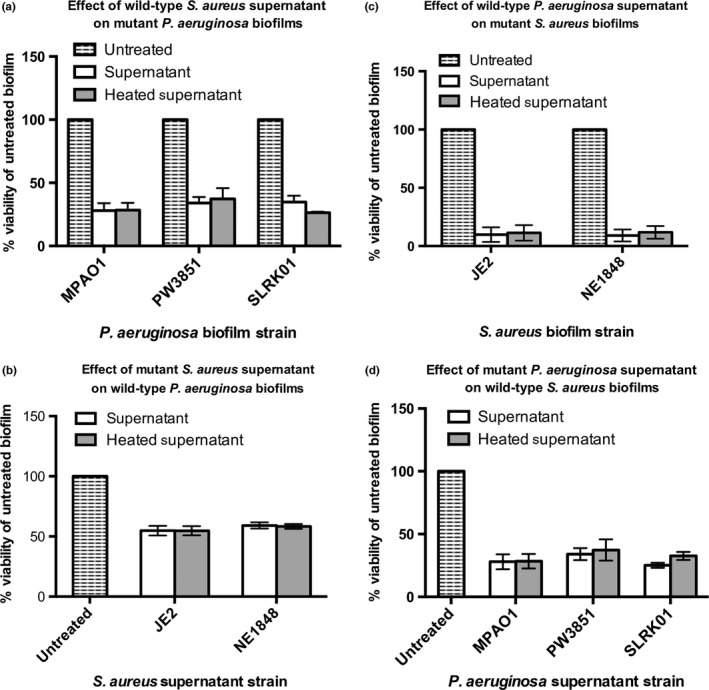
Defects in competition in mixed‐species biofilms cannot be solely attributed to mechanisms of secretion. Contributions of secreted factors from either the mutant or wild‐type strains on the formation of static biofilms of the competitor. Spent media supernatants were acquired from bacterial cultures grown overnight in 20% BHI++ at 37°C shaking, harvested by centrifugation, and filter sterilized. Strains were grown 24 hr at 37°C shaking in 96‐well plate with BHI++ media supplemented with supernatants at a 1:1 ratio. Biofilm viability was determined using the PrestoBlue Cell Viability Reagent read at an excitation and emission wavelengths of 535 nm and 590 nm, respectively (a–d). Defect in competition of mutants is not attributed to the inability of the mutants to combat exoproducts secreted by the wild‐type (either JE2 or MPAO1) competitor (a, b). Defect in competition of mutants is not attributed to the inability of the mutants to secrete exoproducts and inhibit the biofilm viability of competitor in a dual‐species biofilm (c, d). Spent media supernatants were boiled as a control to assess if factors in spent media were protein based. Wild‐type *Staphylococcus aureus* (JE2); *S. aureus lukS‐PV* (strain NE1848); wild‐type *Pseudomonas aeruginosa* (strain MPAO1); *P. aeruginosa PA1595* transposon mutant (strain PW3851); *P. aeruginosa PA1595* clean knockout (strain SLRK01)

Spent media from *S. aureus* planktonic cultures grown for 16 hr was supplemented into the media and biofilm formation was assessed by cellular viability assays. *Staphylococcus aureus* spent media had a dramatic effect on *P. aeruginosa* wild‐type and PW3851 biofilm viability, but no significant difference in viability between PW3851 and wild‐type biofilms was observed (Figure 4a). Similarly, growing *S. aureus* mutant NE1848 in the presence of *P. aeruginosa* spent media demonstrated significant inhibition of biofilm viability in wild‐type and mutant *S. aureus* strains, but no significant viability differences with the mutant when compared to wild‐type *S. aureus* (Figure 4b). Taken together, it is clear that secreted exoproducts from either species do not account for the defects in competition observed with the mutant strain.

#### 3.6.3 The mutant's lack of competitive success cannot be solely attributed to an inability to secrete exoproducts

We hypothesized that the defect in competition may be due to the mutants’ inability to secrete exoproducts. Therefore, we tested the effect of the mutants’ spent media on the biofilm viability of the wild‐type competitor.

**Figure 5:**
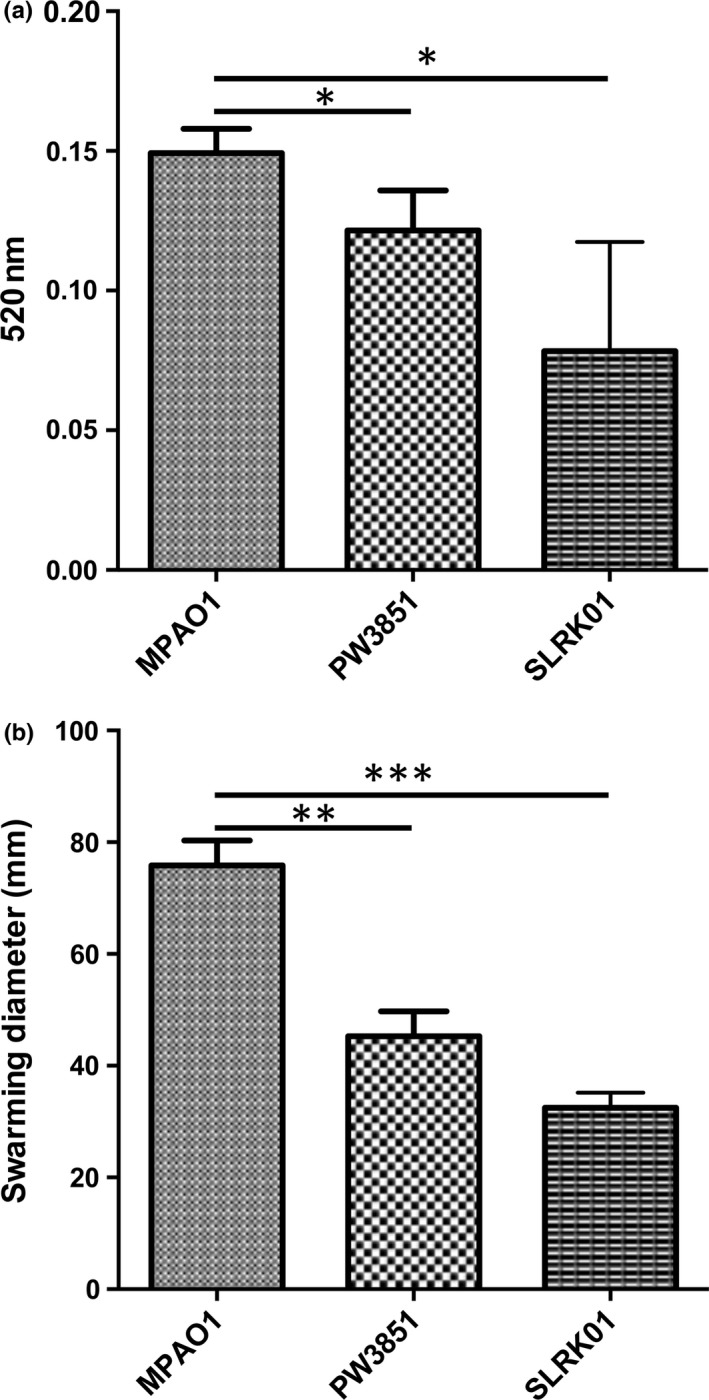
*Pseudomonas aeruginosa PA1595* mutants (PW3851/SLRK01) produce less pyocyanin and have a reduced ability to swarm compared to parental strain. Spent media supernatants of cultures of each strain grown overnight in 20% BHI ++ were extracted with chloroform and HCl and absorbance readings were taken at 520 nm for PYO quantification (a). Swarming motility was assessed for each strain by inoculating 3 μl of a 16 hr overnight culture onto swarm plates (0.5% w/v Bacto agar, 8 g/L of nutrient broth, and 0.5% w/v d‐glucose) and allowed to incubate for 24 hr at 37°C (b). Wild‐type *P. aeruginosa* (strain MPAO1); *P. aeruginosa PA1595* mutant (strain PW3851); *P. aeruginosa PA1595* (strain SLRK01). Data represent the mean ± standard deviation from four independent experiments. Significance was determined by paired Student's *t*‐test (two‐tailed) (*p <* *.05)

We saw strong inhibitory effects of *P. aeruginosa* spent media on *S. aureus* biofilm viability, but no significant difference in *S. aureus* viability when comparing spent media from wild‐type *P. aeruginosa* or PW3851 mutant strain (Figure 4c). No significant difference in *P. aeruginosa* biofilm viability was found using spent media from either *S. aureus* mutant NE1848 when compared to spent media from wild‐type *S. aureus* (Figure 4d). Interestingly, we noted that PW3851 and SLRK01 produce significantly less pyocyanin and have a reduced ability to swarm (Figure 5). Collectively, our results suggest that the observed defects in competition cannot be attributed to the mutants’ inability to secrete exoproducts.

#### 3.6.4 Mechanisms during close interactions contribute to the competitive defects observed with both *S. aureus* mutants

**Figure 6:**
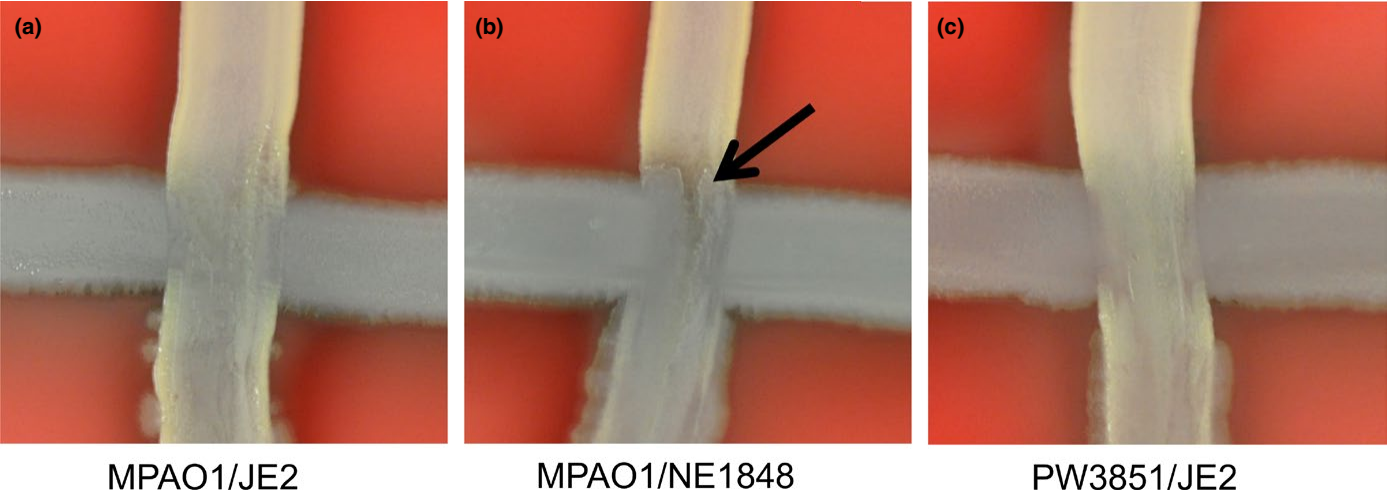
Competitive defects observed with both *Staphylococcus aureus* mutants appear attributable to interactions where *Pseudomonas aeruginosa* and *S. aureus* interface. TSA+ plates were streaked horizontally with overnight cultures of *P. aeruginosa*, allowed to dry, and then streaked vertically with overnight cultures of *S. aureus*. The plates were incubated overnight at 37°C to assess contact inhibition. (a) Wild‐type *P. aeruginosa* (strain MPAO1) was streaked along with wild‐type *S. aureus* (JE2). (b) The zone of interaction show growth of wild‐type *P. aeruginosa* inward at interface when grown with *S. aureus lukS‐PV* mutant (strain NE1848) (black arrow). (c) *Pseudomonas aeruginosa PA1595* mutant (strain PW3851) streaked along with wild‐type *S. aureus* (JE2) look similar to the wild‐type cross‐streaks at the interface. Images are representative of six independent experiments

In order to determine if the lack of competitive success of the mutants could be attributed to mechanisms taking place when the two species are in direct contact, we performed cross‐streak assays of mutant strains with wild‐type competitors. By comparing the zone of interaction (black arrow) when the wild‐type strains were cross hatched (Figure 6a), we noticed that when wild‐type *P. aeruginosa* was grown with NE1848 (Figure 6b), the zones of interaction show more ingrowth of *P. aeruginosa* and an increased area of clearance, suggesting that contact inhibition may be one mechanism for the observed defects when the *S. aureus* mutant was grown with *P. aeruginosa*. When PW3851 and wild‐type *S. aureus* were grown together, the zone of interaction appeared similar to that of the two wild‐type strains (Figure 6c).

### 3.7 Role of sRNAs in mixed‐species biofilms

We hypothesized sRNA regulators contribute to the bacterial physiology coordinating mixed‐species interactions and thus generated sequencing libraries to identify sRNAs differentially expressed in each species when grown in a dual‐species biofilm. Our study assessed sRNAs previously characterized (Table 4) (Eyraud, Tattevin, Chabelskaya, & Felden, 2014; Ferrara et al., 2015; Fozo et al., 2010; Gonzalez et al., 2008; Heurlier et al., 2004; Kay et al., 2006; Park et al., 2013; Sayed, Jousselin, & Felden, 2012; Sonnleitner, Abdou, & Haas, 2009; Wenner, Maes, Cotado‐Sampayo, & Lapouge, 2014; Wilderman et al., 2004), sRNAs previously identified but not characterized in both *P. aeruginosa* (Table S4) and *S. aureus* (Table S5) (Beaume et al., 2010; Ferrara et al., 2012; Gomez‐Lozano et al., 2012; Griffiths‐Jones, Bateman, Marshall, Khanna, & Eddy, 2003; Howden et al., 2013; Kin et al., 2007; Li et al., 2013; Sonnleitner et al., 2008), and novel sRNAs which we functionally categorized in both *P. aeruginosa* (Table S6) and *S. aureus* (Table S7).

**Table 4:** Previously characterized sRNAs differentially expressed in *Pseudomonas aeruginosa* and *Staphylococcus aureus* in mixed‐species biofilm

Common name	Start	End	Strand	Size	Mean RPKM A (MixBiofilm)	Mean RPKM B (Biofilm)	Fold change (A/B)	Adjusted p Value	Other name	Author References
*P. aeruginosa*										
ErsA	6183 500	6183 700	−	201	220 345.77	35 459.30	6.21	5.47E–05	SPA0122, Spot42	Ferrara et al. (2015)
PhrD	785 498	785 570	+	73	3846 804.03	902 778.99	4.26	2.41E–03	SPA0001	Sonnleitner et al. (2008)
RgsA	3318 747	3318 868	+	122	1213.96	4569.60	−3.76	6.44E–03	P16	Livny et al. (2006); Gonzalez et al. (2008); Dotsch et al. (2012); Park et al. (2013)
*S. aureus*										
RsaE	1005 480	1005 540	+	61	2881.56	18 217.54	−6.32	2.04E–32	RsaE, sRNA183, RsaON, Sau20, Teg92, IGR6	Geissmann et al. (2009); Abu‐Qatouseh et al. (2010); Beaume et al. (2010); Howden et al. (2013)
Sau6477	633 395	633 707	−	313	106 860.56	253 874.37	−2.38	9.67E–12	RsaOI, Sau6477, Teg47, sRNA131	Abu‐Qatouseh et al. (2010); Beaume et al. (2010); Bohn et al. (2010); Howden et al. (2013)
RsaA	643 605	643 749	+	145	150 757.93	275 089.95	−1.82	3.06E–06	RsaA, rsaOJ, sau64, Teg88, sRNA132	Geissmann et al. (2009); Abu‐Qatouseh et al. (2010); Beaume et al. (2010); Bohn et al. (2010); Howden et al. (2013); Romilly et al. (2014)
SprX	2088 665	2088 758	−	94	14 742.82	50 240.88	−3.41	8.39E–20	SprX, sRNA299, RsaOR, ssr6,teg15, IGR12	Beaume et al. (2010); Bohn et al. (2010); Howden et al. (2013); Eyraud et al. (2014)
tmRNA	868 406	868 760	+	355	136 365.09	322 914.53	−2.37	1.02E–11	tmRNA, WAN014GIY, Teg150, ssrA, sRNA166	Pichon and Felden (2005); Roberts et al. (2006); Beaume et al. (2010); Liu et al. (2010)
SprA1^a^	1929 827	1929 977	+	151	65 138.68	40 204.19	1.62	4.84E–04	SprA1, IGR1520, Teg8, sRNA285	Pichon and Felden (2005); Roberts et al. (2006); Beaume et al. (2010); Sayed et al. (2012); Howden et al. (2013)
SprA1as	1929 990	1930 044	−	55	7343.69	10733.02	−1.46	2.86E–02	SprA1as, Teg152, sRNA286	Beaume et al. (2010); Sayed et al. (2012); Howden et al. (2013)

a Peptide toxin regulated by *cis*‐antisense sRNA, SprA1as.

Using literature and databases of previously identified sRNAs, eliminating duplicate sRNAs (those with sequence overlap <25 nucleotides), and having a cutoff criteria (*p *>* *.05; fold change >2) demonstrated approximately 100 and 150 sRNAs were differentially expressed in *S. aureus* and *P. aeruginosa*, respectively, during mixed‐ versus single‐species biofilm growth. Interestingly, of these sRNAs in both *P. aeruginosa* and *S. aureus* approximately 25% were upregulated and 75% were downregulated (Table S4 and S5).

#### 3.7.1 Few characterized sRNAs, but many previously identified sRNAs were differentially expressed in *P. aeruginosa* mixed‐ versus single‐species biofilms

Three sRNAs differentially expressed in *P. aeruginosa* during mixed‐ versus single‐species biofilms were previously characterized, with two of them having been functionally characterized (Table 4). ErsA (also annotated as SPA0122) was upregulated approximately sixfold in *P. aeruginosa* in mixed‐species biofilms. ErsA is a sRNA whose transcription is strictly dependent on the envelope stress‐responsive sigma factor σ^22^ (AlgT/U) which is essential for the production of the exopolysaccharide alginate (Ferrara et al., 2015). ErsA has been shown to target the *algC* transcript, is induced upon envelope stress, increases in temperature, and anaerobic conditions and is thought to influence the dynamics of exopolysaccharide biosynthesis underlying the development of biofilm matrix (Ferrara et al., 2015). RgsA (also known as P16) was downregulated almost fourfold in *P. aeruginosa* in mixed‐species biofilms. RgsA's expression is dependent on RpoS, indirectly influenced by response regulator GacA (Gonzalez et al., 2008; Park et al., 2013), conserved across Pseudomonads (Livny, Brencic, Lory, & Waldor, 2006), upregulated in late stationary and biofilm culture conditions, and thought to promote resistance to oxidative stress (Dotsch et al., 2012).

#### 3.7.2 Expression of small RNAs in *S. aureus* involved in capsule formation, amino acid metabolism, and toxin production during biofilm growth with *P. aeruginosa*

RsaA (also annotated as Sau64 and Teg88) is a sRNA that represses translation of the MgrA regulator which activates biofilm production and inhibits capsule formation (Romilly et al., 2014). SprX (also annotated as Teg15 and RsaOR) is a sRNA that also represses capsule formation but through translational inhibition of the *spoVG* operon (Eyraud et al., 2014). RsaA and SprX are downregulated approximately two‐ and threefold, respectively, in *S. aureus* in mixed‐ versus single‐species biofilms (Table 4). RsaE (also annotated as Teg92) is a sRNA that is downregulated 6.3‐fold in *S. aureus* grown in mixed‐species biofilms. RsaE regulates metabolic pathways and increases the amino acid pool by upregulating expression of valine, isoleucine, and leucine operons. SprA1 is a cytolytic peptide toxin that is regulated by a *cis*‐antisense RNA, SprA1_AS_ (also annotated as Teg152) (Fozo et al., 2010; Sayed et al., 2012). Typically, SprA1_AS_ is generated in excess of SprA1 thus inhibiting the translation of this toxin (Sayed et al., 2012). When *S. aureus* is grown in a mixed‐species biofilm SprA1 is upregulated approximately 1.5‐fold, whereas SprA1_AS_ is downregulated approximately 1.5‐fold suggesting an alleviation of translational repression by this antisense RNA and most likely subsequent translation of the SprA1 toxin.

## 4 DISCUSSION

### 4.1 *Pseudomonas aeruginosa* the dominant pathogen: model mimics mixed‐species infections seen in vivo

While it is interesting to study the planktonic interactions of these two species, the biofilm condition is more relevant medically because chronic biofilm infections significantly impair wound healing. In contrast to the trend seen in mixed planktonic cultures where *P. aeruginosa* outcompeted *S. aureus* regardless of media strength, the richness of the media had a strong impact on bacterial numbers in mixed biofilms. Specifically, in 20% BHI++, *P. aeruginosa* outcompeted *S. aureus* (MPAO1/TCH1516 approximately 100:1). In contrast, in 100% BHI++, *S. aureus* outcompeted *P. aeruginosa* (TCH1516/MPAO1 approximately 100:1). We noted that the mixed biofilms imaged by SEM demonstrated these growth trends favoring either *P. aeruginosa* or *S. aureus* depending on the strength of the growth media. It is interesting to speculate why *S. aureus* is a better competitor in the rich growth medium. Perhaps the stress of minimal media induces the expression of various harmful components by *P. aeruginosa*, such as pyocyanin or siderophores, which could inhibit the growth and success of *S. aureus*. Additionally, we have observed a more rapid doubling time of *S. aureus* in planktonic culture. Combined with the abundance of nutrients, the ability to replicate more quickly may explain the ability of *S. aureus* to outcompete *P. aeruginosa* in high‐energy environments. Overall, biofilms grown in 20% BHI++ and harvested from the drip‐flow reactors resulted in bacterial burdens seen in in vivo studies and clinical infections and thus represented an appropriate model to study bacterial interactions in mixed‐species biofilms using RNA‐seq.

### 4.2 *Pseudomonas’* competitive advantage through normal growth and biofilm development processes

*Pseudomonas aeruginosa* differentially expressed relatively few ORFs (~0.3% of the genome) when grown in a mixed‐ versus single‐species biofilm, in contrast to *S. aureus* which had a more robust transcriptomic response showing nearly 150 up‐ and downregulated ORFs (~5% of the genome). A similar trend was observed in a recent study comparing *P. aeruginosa* strain PA14 and *S. aureus* strain 8325–4 in an in vitro cystic fibrosis (CF) model (Filkins et al., 2015). Therefore, *P. aeruginosa* appears to easily maintain itself as a dominant organism in various in vitro systems. Interestingly, most mutants lacking an ORF that was upregulated in *P. aeruginosa* in mixed‐species biofilms were defective for growth or biofilm development and thus their contributions during mixed‐species biofilms could not be assessed. Taken together, this suggested to us that *P. aeruginosa* may play a more passive role in mixed‐species biofilms and its success appears to be a result of the ability to carry out normal growth and biofilm developmental processes.

Passive killing of competitors in the niche may be a result of intrinsic events required for biofilm development and maturation. One of these intrinsic events is bacterial autolysis, a form of programmed cell death that releases lytic enzymes and bacteriocins to which competitors may be sensitive to (Michel‐Briand & Baysse, 2002). Holin/antiholin systems are responsible for controlling autolysis which releases lytic enzymes and cellular material that is then utilized to build structural components of the biofilm, foster specific biofilm architecture, and promote dispersion (Ma et al., 2009). One of the few ORFs upregulated in *P. aeruginosa* during mixed‐species biofilm growth is *PA3431* and *PA3432*. *PA3432* and *PA3431* are *lrgA*/*cidA* and *lrgB*/*cidB* homologs, respectively, and are holins or antiholins that can coordinate and control the exact timing of autolysis. This autolysis may be triggered by nutrient deprivation, stress, or insults from a competitor. In our SEM analyses both monospecies *P. aeruginosa* and mixed biofilms showed an increased level of extracellular material when grown in 20% BHI++ under static conditions. In contrast, there is little, if any, of this material present in *S. aureus* biofilms grown in 20% BHI++ or in *P. aeruginosa* biofilms grown in 100% BHI++, or in the drip‐flow reactor. As 100% BHI++ and growth in the drip‐flow reactor provide a rich growth environment, these results suggest that *P. aeruginosa* is responsible for making this extracellular material under nutrient limiting conditions, and this material may be a byproduct produced by autolytic events required for biofilm development. In addition, we have observed that *P. aeruginosa* biofilms are significantly more mucoid and will form at the air–liquid interface more readily than those produced by *S. aureus*, perhaps partly as a result of the extracellular material visualized using SEM (Figure 2). Ultimately, autolysis by *P. aeruginosa* may provide a competitive advantage through the inadvertent release of lytic enzymes and bacteriocins contributing to subsequent growth inhibition of *S. aureus*.

Numerous studies have shown that *P. aeruginosa* can actively inhibit or kill competitors in the niche, including *S. aureus*. *Pseudomonas aeruginosa* attacks its competitors indirectly through the production of secreted exoproducts and directly by injecting toxins into competitors by the Type III and Type VI secretion system (T3SS, T6SS) (Russell et al., 2013). Although Park et al. (2012) showed that *P. aeruginosa* secretes an antibiofilm protease encoded by *lasB* against *S. aureus* (Park et al., 2012), we did not see *lasB* upregulated in *P. aeruginosa* when grown in the presence of *S. aureus*. Also, in *P. aeruginosa* in mixed‐species biofilms there was no significant change in expression of T6SS ORFs and *exoS*, a Type III secreted toxin, was downregulated which further suggested that in our study *P. aeruginosa* did not mount a defensive response against *S. aureus* during biofilm growth and competitive advantage might be obtained by carrying out normal growth and biofilm process.

### 4.3 Contributions of a novel *P. aeruginosa* ORF during competition with *S. aureus*

Our studies did reveal a novel ORF that provides a competitive advantage to *P. aeruginosa* in mixed‐species biofilms with *S. aureus*. PA1595 is a hypothetical protein predicted to be in an operon with two previously uncharacterized ORFs (*PA1593* and *PA1594*). Cluster of orthologous group (COG) predictions of both PA1593 and PA1594 demonstrated significant similarities to HGG motif‐containing thioesterase proteins and possible involvement in aromatic compound catabolism. Conserved domain database (CDD) predictions further suggested that both PA1593 and PA1594 contain domains indicative of phenylacetic acid (PAA) degradation. PAA serves as an antipathogenic factor released at stationary phase and plays a role in inhibiting quorum sensing. Musthafa, Sivamaruthi, Pandian, and Ravi (2012) demonstrated that exogenous addition of PAA leads to poor swimming motility and reductions in QS‐dependent pyocyanin, exopolysaccharide, protease, and elastase production in PAO1 (Musthafa et al., 2012). Our results demonstrate that a *PA1595* transposon mutant strain (PW3851) and a clean knockout (SLRK01) were both defective during competition with *S. aureus*. Neither the supernatant of MPAO1, PW3851, or SLRK01 had a significant effect on *S. aureus* biofilm viability, but interestingly both *PA1595* mutant strains, PW3851 and SLRK01, produced significantly less pyocyanin and had a reduced ability to swarm. Although PAA levels were not assessed in our study, the role of PA1595 is most likely dynamic and our work begins to unravel its contributions for success of *P. aeruginosa* in mixed‐species biofilms.

### 4.4 *Staphylococcus aureus* has a robust genomic response to growth with *P. aeruginosa* suggestive of increased virulence and capsulation, with a concomitant decrease in biofilm formation

Despite *S. aureus* being significantly outcompeted by *P. aeruginosa*, it is well known that *S. aureus* is a particularly virulent bacterium, able to cause severe disease, impair wound healing, and has numerous virulence factors (Pastar et al., 2013; Seth, Geringer, Galiano, et al., 2012; Seth, Geringer, Hong, et al., 2012). Similar to previous reports, *S. aureus* upregulates a few of these virulence genes in response to being grown with *P. aeruginosa*, including both ORFs encoding leukocidin (*lukS‐PV* and *lukF‐PV*) and α‐hemolysin (*hla*) (Pastar et al., 2013). Contrary to a previously published report that saw a downregulation of *spA* in a mixed‐species biofilm in vivo (Pastar et al., 2013), we saw significant upregulation of *spA* in our in vitro conditions and these differences in expression may be a result of disparities in experimental conditions.

However, our global study allowed insight into myriad of other factors at play and interestingly, several *S. aureus* genes known to be important for biofilm formation including fibronectin‐binding proteins A and B (FnbAB), clumping factor B (ClfB), and coagulase (Coa) (Abraham & Jefferson, 2012; Friedrich et al., 2003; McCourt et al., 2014; Mulcahy et al., 2012; O'Brien et al., 2002; Zapotoczna et al., 2015) were downregulated when *S. aureus* was grown in a biofilm with *P. aeruginosa*.

Also, when *S. aureus* was grown in a biofilm with *P. aeruginosa*, 13% and 45% of the downregulated ORFs were those involved in purine and pyrimidine biosynthesis and amino acid transport and metabolism, respectively, while 15% of the upregulated ORFs were involved in capsular biosynthesis, though it has been shown that these strains of *S. aureus* do not actually form a capsule due to mutations in various capsule ORFs (Boyle‐Vavra et al., 2015). As shown by Filkins et al., 2015; the presence of *P. aeruginosa* exoproducts can drive *S. aureus* from aerobic to lactic acid fermentation (Filkins et al., 2015). We noted similar upregulation of fermentation related ORFs, including *pflAB*,* ldh*, and *adh*. Taken together, it is plausible that *S. aureus* increases production of specific virulence factors, downregulates biofilm formation, relies on alternative respiration in order to coexist in this competitive niche with the dominant pathogen, *P. aeruginosa*.

### 4.5 Contributions of specific *S. aureus* ORFs during competition with *P. aeruginosa*

As previously mentioned, *S. aureus* is able to successfully colonize biotic and abiotic surfaces and persist in chronic wound environments by avoiding killing by the host immune system. Many *S. aureus* strains produce a polysaccharide capsule that promotes abscess formation and prevents phagocytosis by polymorphonuclear leukocytes (PMNs) (Herbert et al., 1997; Karakawa, Sutton, Schneerson, Karpas, & Vann, 1988; Kiser, Cantey‐Kiser, & Lee, 1999). Of the ORFs upregulated in *S. aureus* in mixed‐ versus single‐species biofilms, 15% corresponded to type 5 capsule (*cap5EF*,* cap5H‐P*). Although this strain is known to not form a capsule due to specific mutations in various capsule ORFs (Boyle‐Vavra et al., 2015), others strains do, and it is possible that upregulation of capsule could serve as a protective mechanism for those strains when they are faced with adverse conditions such as host immune responses and competition with other microbial species.

Panton–Valentine leukocidin (PVL) is an important virulence factor implicated in severe pneumonia and necrotizing fasciitis (Boyle‐Vavra & Daum, 2007) and is composed of two subunits (*lukF‐PV* and *lukS‐PV*) that combine to form pores, particularly in the membranes of PMNs. While ORFs encoding PVL are upregulated in mixed infections (Pastar et al., 2013), it is unknown what role PVL plays in *S. aureus*’ competitive success during mixed‐species growth. We saw that a *lukS‐PV* transposon mutant strain (NE1848) was defective for competition with *P. aeruginosa* and sought to identify if PVL was harmful to not only host PMN cells, but also bacterial cells. Subjecting *P. aeruginosa* to spent media isolated from NE1848 showed no significant change in biofilm viability, though growing NE1848 in cross‐hatch assays with *P. aeruginosa* suggesting that PVL may play a role in *S. aureus*’ competitive success when *S. aureus* is in close proximity to a competitor such as *P. aeruginosa*.

RNA‐seq and analysis of ORFs makes it clear that *S. aureus* responds strongly when grown in the presence of *P. aeruginosa*. While CI numbers show that *P. aeruginosa* outcompeted *S. aureus*,* S. aureus* mounts a robust genetic response, upregulating various virulence factors and converting to lactic acid fermentation while downregulating ORFs involved in basic metabolism and biofilm formation. Increased virulence and capsulation (for capsulated strains) are likely strategies that *S. aureus* adopts in order to prevent its total elimination from a mixed‐species biofilm.

### 4.6 sRNAs parallel genomic response in mixed‐species biofilms

Because sRNAs can target mRNAs and regulate their translation without influencing the transcriptional output, we speculate that this phenomenon may be occurring in *P. aeruginosa* to dictate a phenotype providing a competitive advantage in mixed‐species biofilms. ErsA was upregulated in *P. aeruginosa* during mixed‐species biofilm growth and is a sRNA that is induced upon envelope stress. It is interesting to speculate how ErsA may help counteract any agents *S. aureus* is producing. On the other hand, ErsA is involved in regulating the dynamic state of the exopolymeric substance and may be expressed to coordinate efforts required for typical biofilm maturation. We suspect from the ORF RNA‐seq analyses that *P. aeruginosa* is minimally affected by the presence of *S. aureus* after the establishment of a biofilm. In support of this, RgsA, a sRNA that has been shown to help resist against oxidative stress, was downregulated in *P. aeruginosa* in mixed‐species biofilms. Nevertheless, numerous uncharacterized sRNAs were differentially expressed and our study begins to help define their roles in the pathobiology of *P. aeruginosa*.

RsaA and SprX are two small RNAs known to repress capsule formation and were downregulated in *S. aureus* in mixed‐ versus single‐species biofilms. This is consistent with our RNA‐seq analyses of ORFs because approximately 15% of upregulated ORFs in *S. aureus* in mixed‐ versus single‐species biofilms were those involved in capsule formation. 80% of *S. aureus* isolates recovered from infected patients are encapsulated (Cocchiaro et al., 2006), therefore capsules must also be an important tool against or protected from bacterial competitors such as *P. aeruginosa*. Furthermore, RNA‐seq assessing small RNAs demonstrated that *S. aureus* induces expression of a type I toxin‐antitoxin (TA) module (SprA1/SprA1_AS_) when grown as a biofilm in the presence of *P. aeruginosa*. SprA1 toxin's translation is repressed by the *cis*‐antisense RNA, SprA1_AS_. SprA1 was expressed approximately 10 times higher than SprA1_AS_ in *S. aureus* in mixed‐species biofilms suggesting that this toxin is being expressed. SprA1 is a cytolytic peptide that is autolytic, kills eukaryotic cells, as well as Gram‐positive and ‐negative bacteria (Fozo et al., 2010; Sayed et al., 2012). Autolytic TA systems are involved in enriching the population of persister cells because metabolically dormant cells are refractory to the toxins whereas rapidly growing siblings are sensitive. This suggests that *S. aureus* may undergo this altruistic behavior in effort to coexist and combat *P. aeruginosa* in mixed‐species biofilms. Finally, we saw a downregulation of RsaE, a sRNA regulator of metabolism which activates amino acid biosynthesis, and is most likely contributing to the decrease in amino acid biosynthesis pathways indicated by our RNA‐seq analyses of ORFs. Collectively, our results suggested that the sRNA profile closely mimics the trends seen when the ORFs were analyzed, and expression of small RNAs appears to steer *S. aureus’* phenotype toward capsule formation, decreased amino acid metabolism, and production of toxins that promote persistence during biofilm growth with *P. aeruginosa*.

## 5 CONCLUSION

The advent of deep sequencing (RNA‐seq) has allowed researchers to identify the physiological contributions of regulatory small RNAs and thus our work utilized RNA‐seq to identify the entire genomic response, both ORFs and small RNAs, required for the success of each pathogen in a mixed‐species biofilm. Our methods aimed to recapitulate the dynamic nature of a mixed‐species biofilm seen in in vivo infections. Based on our results we speculate that during an acute (planktonic) mixed‐species infection, there is a robust response or active combat from both pathogens until a state of equilibrium is reached during the maturation of a chronic infection (biofilm). Ultimately, one organism dominates in the environment (*P. aeruginosa*) and proceeds by carrying out the typical operations necessary for establishing a biofilm community crowding out, but not completely eliminating its competitor (*S. aureus*). It is likely that combating these infections may necessitate a reactivation or tip in this equilibrium. Also, our work suggests that sRNAs are coordinating with the genomic response to carry out the adaptive changes necessary to attain a homeostatic mixed‐species biofilm.

## CONFLICT OF INTEREST

None declared.
