# Supplementary material for: Global transcriptome responses including small RNAs during mixed‐species interactions with methicillin‐resistant Staphylococcus aureus and Pseudomonas aeruginosa
Source: Microbiologyopen. 2016 Nov 21;6(3):e00427. doi: 10.1002/mbo3.427 (PMC5458535; doi:10.1002/mbo3.427)
Supplement: Supplementary file 1 [file MBO3-6-na-s001.docx]

**
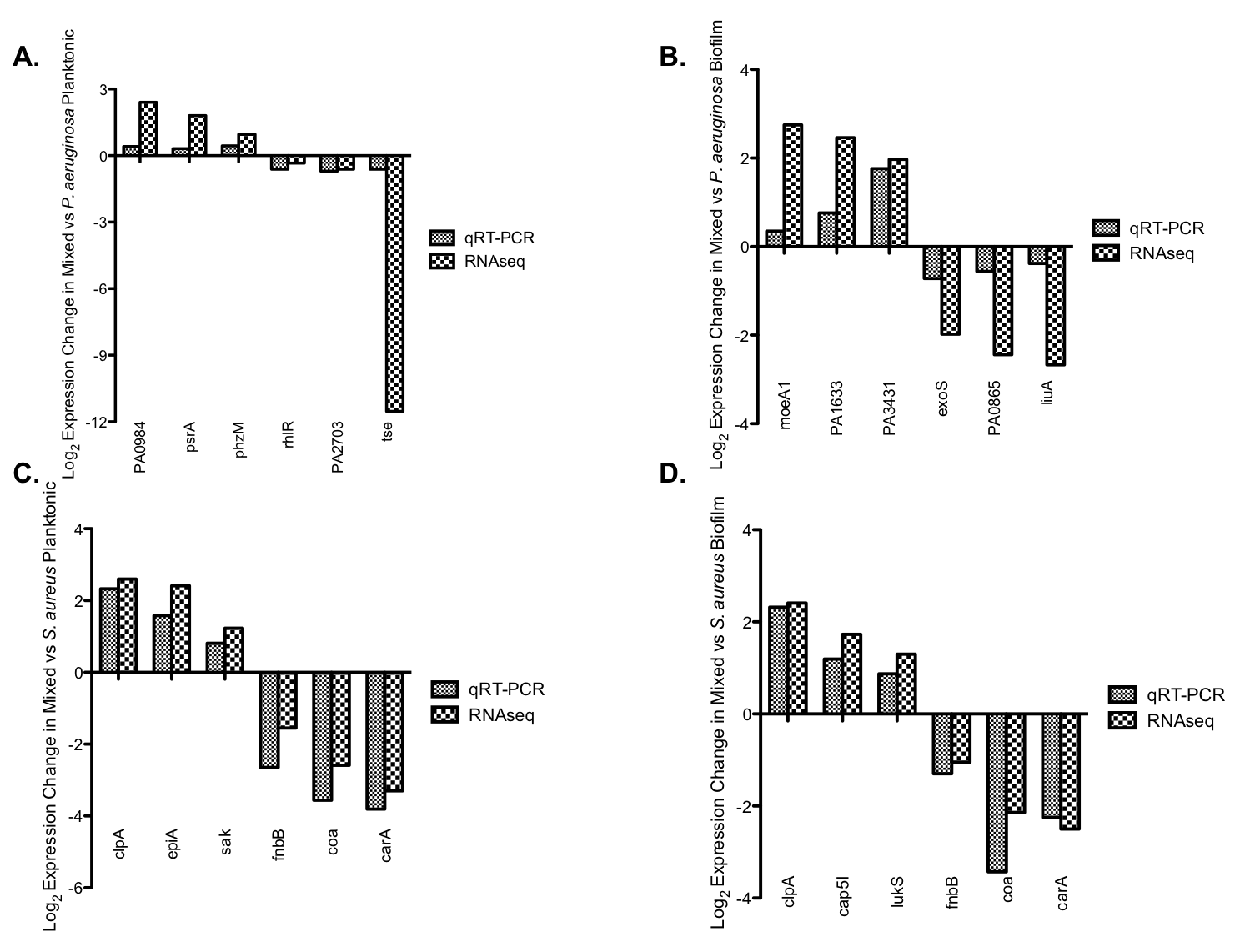
**

**Fig. S1.  Quantitative real-time PCR analysis confirms trends from RNA sequencing data.**  RNA extracted from biofilms and planktonic cultures was converted to cDNA using the iScript Select cDNA Synthesis Kit.  cDNA was subjected to quantitative real-time PCR using SYBR Green PCR master mix with a final concentration of 0.3 µM of oligonucleotides (Table S1) using the ABI Prism 7300 system.  Gene expression levels were compared for selected hits generated from RNA sequencing results with those selected hits determined by quantitative PCR between mixed- versus single-species *P. aeruginosa* planktonic **(A)** and biofilm **(B)** and *S. aureus* planktonic **(C)** and biofilm **(D)** cultures.  Data were normalized to expression levels of *gmk* for *S. aureus* RNA and *fabD* for *P. aeruginosa* RNA and fold differences were generated using the 2^-ΔΔCt^ method.


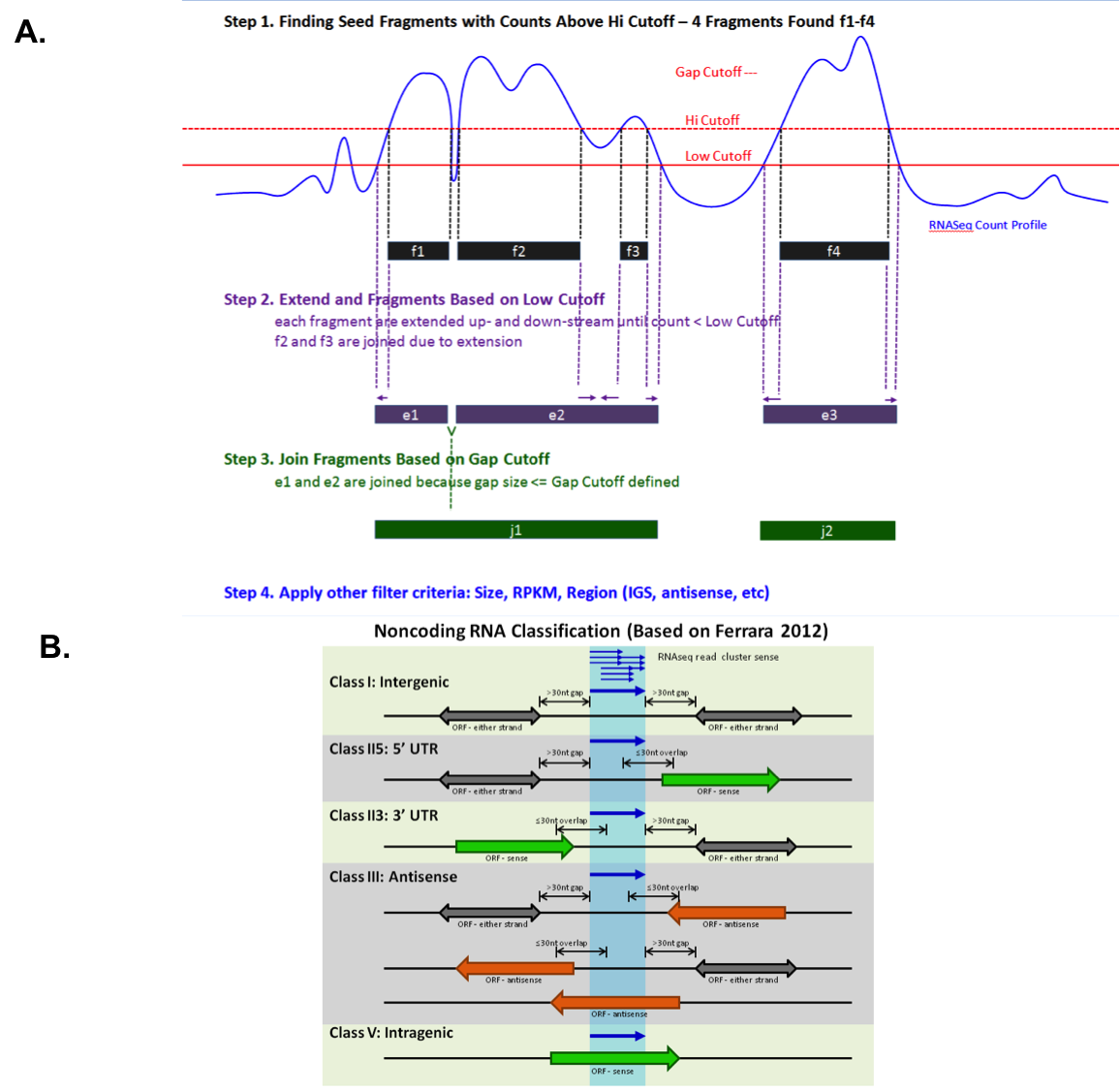
 **Fig. S2.  Schematic describing small RNA detection and categorization.**  **A.** Diagram and flow chart for the detection of small RNAs and the quantification of the reads per kilobase of transcript per million mapped reads (RPKMs). **B.** Classification of small RNAs into various classes based on their location on the chromosome.
